# Resveratrol and Its Natural Analogs Mitigate Immune Dysregulation and Oxidative Imbalance in the Endometriosis Niche Simulated in a Co-Culture System of Endometriotic Cells and Macrophages

**DOI:** 10.3390/nu16203483

**Published:** 2024-10-14

**Authors:** Agata Gołąbek-Grenda, Wojciech Juzwa, Mariusz Kaczmarek, Anna Olejnik

**Affiliations:** 1 Department of Biotechnology and Food Microbiology, Poznan University of Life Sciences, 48 Wojska Polskiego St., 60-627 Poznan, Poland; agata.golabek@up.poznan.pl (A.G.-G.); wojciech.juzwa@up.poznan.pl (W.J.); 2 Department of Cancer Immunology, Poznan University of Medical Sciences, Garbary 15 St., 61-866 Poznan, Poland; markacz@ump.edu.pl; 3 Gene Therapy Laboratory, Department of Cancer Diagnostics and Immunology, Greater Poland Cancer Centre, Garbary 15 St., 61-866 Poznan, Poland

**Keywords:** endometriosis, inflammation, macrophages, co-culture, transwell system, oxidative stress, resveratrol

## Abstract

**Background:** Inflammation and immune cell dysfunction are critical facilitators of endometriosis pathophysiology. Macrophages are renowned for stimulating lesion growth, vascularization, innervation, and pain generation. By combining macrophages and endometriotic cells, we determined if resveratrol and its natural analogs can target the immune dysregulation and oxidative imbalance in endometriosis. **Methods:** After treatment with compounds (5, 10, 25 µM), we evaluated the expression of key inflammatory and oxidative stress markers, cytokines release, and ROS production by applying q-PCR, ELISA, Cytometric Beads Array, and multiplexed fluorogenic staining and flow cytometry analysis with bioimaging. **Results:** The results showed that endometriosis-related macrophages treated with stilbenes have impaired expression of pro-inflammatory markers (*IL6*, *IL8*, *IL1B*, *TNF*, *CCL2*, *CXCL10*, *PTGS2*). The effect of resveratrol, pterostilbene, and piceatannol was observed, especially in reducing *IL1B*, *CCL2*, and *CXCL10* genes up to 3.5-, 5-, and 7.7-fold at 25 µM, respectively. Also, with piceatannol or polydatin exposure, the IL-6 decrease was noticeable. This study reported an antioxidant effect by reducing ROS-positive cells from 96% to 48% by pterostilbene. Results from flow cytometry correlated with the transcript activation of detoxification enzymes (*SOD*, *GPX*). **Conclusions:** Prospects for potential therapy based on regulating the immune microenvironment and reducing the accumulation of free radicals with stilbenes application were described in the article.

## 1. Introduction

Endometrial macrophages play key roles in mediating the menstrual cycle through the initiation of the breakdown of endometrium during menstruation [1] or contribution to tissue remodeling during the establishment of pregnancy [2]. Macrophages exhibit extensive plasticity, and their phenotype and function are highly specific to the local tissue milieu. Dysregulation of their activity occurs in abnormalities and pathologies of the uterus, including endometriosis. It is evident that macrophages are abundant in endometrial lesions and are critical for their establishment and growth. Their enhanced ability to produce pro-inflammatory cytokines contributes to a microenvironment that favors pelvic inflammation and promotes other immune cells’ recruitment, endometriotic cells’ proliferation, angiogenesis, and innervation, as well as leads to the generation of endometriosis-associated pain [3,4]. Therefore, the reciprocal communication between macrophages and endometriotic cells appears, and their intercellular dialogue sustains the formation of endometriosis lesions. As macrophages are the predominant cellular component in endometriotic tissue, the targeting of macrophage alternation could be critical in developing potential therapeutic candidates.

Resveratrol is a phenomenal natural compound commonly occurring in many plant species, including coarse cereals, potatoes, beans, fruits, and nuts [5]. It became well known due to its anti-cancer potential and promising anti-inflammatory, anti-oxidant, and anti-microbial properties investigated in extensive research [6]. Recently, it was reported that grapes do not serve as the richest source of resveratrol, but rather that the richest sources are tangerine, peach, walnut, and sweet potato. Obtaining resveratrol in the daily diet from everyday food items is worth encouraging, as red wine should not be the primary source of resveratrol due to the harmful impact of alcohol on human health. Recent studies have indicated that the current daily resveratrol intake, estimated at 0.7 mg/day, is insufficient to achieve the health-beneficial effects [5]. Therefore, there is a definite need to increase resveratrol intake to 30–150 mg/day and improve its bioavailability through the development of breeding techniques, consuming foods enriched in resveratrol as well as improvement of extraction methods, and utilization of innovative drug-delivery methods in nanoparticle structures [5].

In searching for potential health-beneficial agents with increased bioavailability, it is necessary to investigate the biological mechanisms for which resveratrol analogs and metabolites can reach the target tissues and exert biological activity. Experiments utilizing analogs demonstrated that resveratrol hydroxylation to piceatannol (3,5,3′,4′-tetrahydroxy-trans-stilbene) attributed the compound’s antioxidant activity and growth-inhibitory effects [7]. This plant-derived stilbene is found in peanuts, teas, berries, and passion fruit. Passion fruit is a rich piceatannol source in its seed, detected at a 4.8 mg/g concentration. Although the benefits of piceatannol have not been studied as extensively as resveratrol, studies have shown a range of health-promoting biological properties with an emphasis on antileukemic, antiangiogenic, and cardioprotective potential, as well as promoting fat metabolism [8]. A recent randomized, double-blind, placebo-controlled crossover comparison study found that consuming 10 mg of piceatannol daily from passion fruit seeds for 7 days increases fat burning in healthy subjects [9]. Pterostilbene (3,5-dimethoxy-4′-hydroxystilbene), with two extra methoxy groups in its structure, shows greater lipophilicity than the parent compound and is, therefore, characterized by higher intestinal permeability, cellular absorption, and increased stability [10]. Pterostilbene is mainly found in blueberries and *Vaccinium* berries up to 500 ng/g. The amount of pterostilbene in dietary sources appears insufficient to exert health benefits. Therefore, formulating pure compound supplements or employing strategies like transgenic alteration or metabolic engineering offers a method to provide nutritional levels. Currently, several clinical trials for pterostilbene are being undertaken, e.g., in treating acute kidney injury and improving vascular endothelial functions and cardiovascular health. In clinical studies, dietary blueberry supplements or combining pterostilbene with other drugs, mainly at 25–125 mg doses, are applied [11]. Polydatin (3,4′,5-trihydroxystilbene-3-β-D-glucoside), in its glycoside resveratrol form, possesses different biological characteristics, such as greater bioavailability [12,13]. The reported therapeutic targets of polydatin include many that have beneficial biological activities against cancers, infections, cardiovascular diseases, diabetes, and gastric and hepatic failure [14]. Grapes, mulberries, cocoa products, peanuts, and hop flowers (*Humulus lupulus*) are prominent dietary sources of polydatin. However, polydatin was primarily isolated from the rhizome *Polygonum cuspidatum* at amounts of up to 14.43 mg/g. Polydatin has been evaluated in different clinical trials involving women’s diseases and gastrointestinal and liver diseases. The effectiveness was achieved in a 40–600 mg dose range per day [14,15]. A combination of polydatin and palmitoylethanolamide impaired inflammatory factors and exhibited analgesic effects on endometriosis in several in vivo and clinical studies [14].

Natural bioactive compounds can target pathological processes involved in endometriosis, like altered inflammatory microenvironment and oxidative stress [6]. Numerous studies suggest that resveratrol demonstrated an immunomodulatory role in immunologic disorders, like cancers, autoimmune, neurodegenerative, metabolic, cardiovascular, and infectious diseases [16,17]. Regarding immune cells, it has been found that resveratrol affects the anti-inflammatory profile in macrophages, exhibits inhibitory function on T cells, reduces CD4+CD25+ regulatory T cells’ suppressive function, and stimulates the killing activity of NK cells. Studies on macrophages revealed that resveratrol regulates TLR-mediated inflammatory responses, nuclear factor kappa B (NF-κB) activation, and cyclooxygenase-2 (COX-2) expression. It attenuated TLR4-TRAF6, mitogen-activated protein kinase (MAPK), and AKT pathways in lipopolysaccharide (LPS)-stimulated macrophages. Resveratrol reduced granulocyte-macrophage colony-stimulating factor (GM-CSF) production, decreased oxidative LPS effect, and modulated immune response linked to prostaglandin E2 (PGE2) and Sirtuin 1 (SIRT1) level [16,18]. Since endometriosis is an inflammatory pathology, this study aims to investigate the comparative anti-inflammatory effects of resveratrol, pterostilbene, piceatannol, and polydatin on endometriosis. It is suggested that macrophage influence on endometriotic cells may be fundamentally important for endometriosis development; however, the cell interactions have not been fully understood. Therefore, this study investigated the effect of stilbenes in the co-culture model of macrophages and endometriotic cells. We analyzed the expression of key inflammatory and oxidative stress markers, production of cytokines, and radical scavenging activity in the activated endometriosis-related macrophages. We also discussed whether the co-culture system could imitate the inflammatory niche in endometriosis.

## 2. Materials and Methods

### 2.1. Chemicals

**Figure 1:**
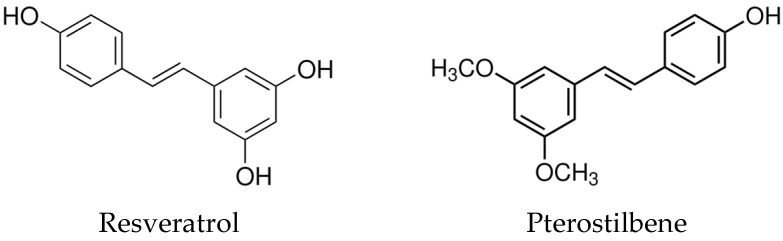

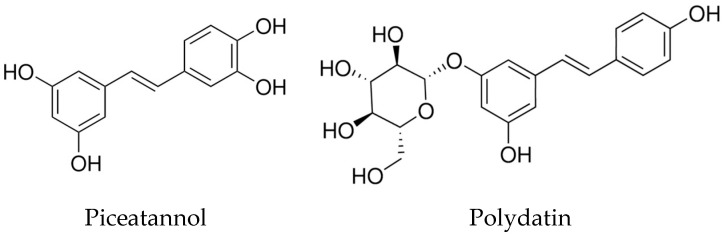
Chemical structures of resveratrol, pterostilbene, piceatannol, and polydatin.

Unless stated otherwise, all chemicals were purchased from Merck KGaA (Darmstadt, Germany). Resveratrol and its analogs were also supplied by Merck KGaA as a solid form and with a high purity (resveratrol ≥ 99%; pterostilbene ≥ 97%; piceatannol ≥ 98%; polydatin ≥ 98.0%). Trans isomers of compounds were used in this study (Figure 1). The stock solution of resveratrol, pterostilbene, piceatannol (80 mM), and polydatin (40 mM) was prepared by dissolving in 96% alcohol and stored at −80 °C. Working solutions (5 mM, 2 mM, 1 mM) were prepared immediately before experiments by diluting the stock solution in 96% alcohol. Then, they were added to the cell culture medium, maintaining 0.5% alcohol concentration in each culture.

### 2.2. Cell Cultures

Immortalized human endometriotic epithelial cells (12Z) were obtained from Applied Biological Materials Inc. (Richmond, BC, Canada). They were cultured in Dulbecco’s Modified Eagle Medium combined with Nutrient Mixture F-12 (DMEM/F12) supplemented with fetal bovine serum (FBS, 10%) and gentamycin (50 mg/L). Human monocyte THP-1 cells (ATCC TIB-202) were purchased from American Type Culture Collection (ATCC, Manassas, VA, USA) and cultured in RPMI 1640 (Invitrogen, Waltham, MA, USA) containing FBS (10%), gentamycin (50 mg/L), and 2-mercaptoethanol (0.05 mM). THP-1 cells were maintained at a minimum density of 2 × 10^5^ cells/mL and subcultured when their concentration reached 8 × 10^5^ cells/mL. They were passaged every 48 h by adding a fresh medium or complete medium replacement until the culture reached appropriate cell density. Both cell lines were grown at 37 °C under an air (95%)/CO_2_ (5%) atmosphere. During the experiments, they were regularly tested regularly for mycoplasma contamination.

### 2.3. Macrophages Differentiation and Co-Culture Setup

**Figure 2:**
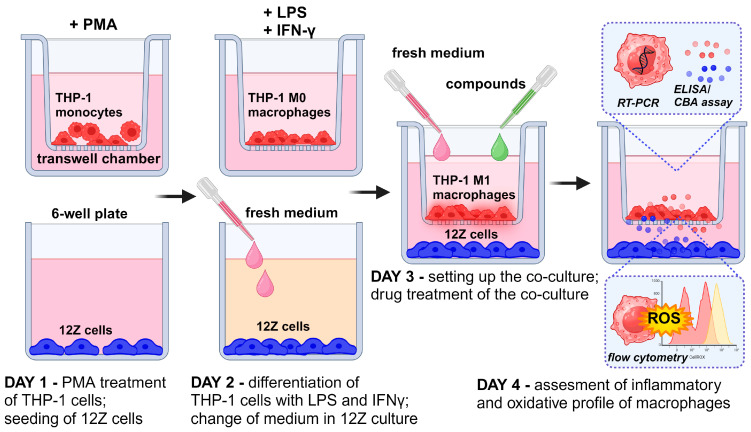
Schematic representation of research design workflow.

Monocytes were stimulated with 10 ng/mL PMA (phorbol-12 myristate 13-acetate) for 24 h, followed by washing with RPMI 1640 to eliminate the effect of PMA, as described by Smith et al. [19]. Morphological changes in cell size, pseudopodia formation, and cell adhesion were observed to assess the acquisition of a macrophage-like phenotype. The co-culture system was prepared in 6-well hanging cell culture inserts (0.4 µm pore size; Merck KGaA, Darmstadt, Germany). THP-1 monocytes were seeded (2 × 10^5^ cells/cm^2^) into the upper compartment of the transwell system in 2 mL of RPMI 1640 medium supplemented with 10% FBS and placed into a 6-well plate containing 3 mL of medium. After seeding, THP-1 cells were treated immediately with PMA (10 ng/mL) for 24 h to stimulate differentiation. A new 6-well plate was prepared the same day with 12Z cells, seeded at 0.3 × 10^5^ cells/cm^2^ in DMEM/F12 with 10% FBS. PMA-activated THP-1 cells (in the insert) and 12Z cells (in the 6-well plate) were incubated separately for 24 h for their attachment. The next day, the differentiated THP-1 cells were washed with the medium. Macrophages were polarized to M1 macrophages with 50 ng/mL IFN-γ (PeproTech, Cranbury, NJ, USA) and 10 pg/mL LPS (PeproTech, Cranbury, NJ, USA) in RPMI 1640 medium supplemented with 10% FBS according to the method described by Smith et al. and Genin et al. [19,20]. Simultaneously, the medium in the 6-well plate with 12Z cells was changed to RPMI 1640 with 2% FBS. After 24 h, the chambers containing THP-1-derived macrophages were hung on the 6-well plates with the 12Z cells to obtain a co-culture cell system. Macrophages and 12Z cells were co-cultured in RPMI 1640 medium with 2% FBS and treated with resveratrol and its derivatives for 24 h. Figure 2 presents a schematic experimental design that includes macrophage differentiation, macrophage polarization, a co-culture setup, treatment with analyzed compounds, and further analysis.

### 2.4. Cytotoxicity Assay

THP-1 cells were cultured in 24-well plates at an initial density of 1 × 10^5^/cm^2^ and stimulated by 24 h incubation with 10 ng/mL PMA. The cultures were then treated with resveratrol at 1 µM to 50 µM and incubated for 24 h under standard culture conditions in RPMI 1640 medium and 2% FBS. The 12Z cells were grown in 24-well plates at an initial density of 0.25 × 10^5^/cm^2^ and treated with resveratrol at the same concentration range and under the same culture conditions. Cell viability and metabolic potential in both cultures were determined using the colorimetric MTT (3-(4,5-dimethylthiazol-2-yl)-2,5-diphenyltetrazolium bromide) assay following the method described previously [21]. Absorbance was measured with a Tecan M200 Infinite microplate reader (Tecan Group Ltd., Männedorf, Switzerland).

### 2.5. RNA Extraction and Real-Time PCR

Total RNA from THP-1-derived macrophages co-cultured with 12Z cells was extracted using Trizol reagent (Invitrogen, Waltham, MA, USA) according to the previously described protocol [22]. Complementary deoxyribonucleic acids (cDNAs) were synthesized using the cDNA Transcriptor First-Strand kit (Roche Diagnostics GmbH, Penzberg, Germany). Quantitative real-time polymerase chain reaction (qRT-PCR) was performed using SYBR^®^ Select Master Mix (Life Technologies, Carlsbad, CA, USA). The PCR reaction included 1 µL cDNA, 1 µL of specific forward and reverse primers (5 µM), 12.5 µL SYBR^®^ Select Master Mix, and nuclease-free water in the amount to a final volume of 25 µL. The PCR reaction mixture was denatured at 94 °C for 10 min, followed by 40 cycles of 95 °C for 40 s, 59 °C for 30 s, and 72 °C for 30 s. The relative mRNA expression was calculated using the 2^−ΔΔCt^ method, where Ct represents the threshold cycle. GAPDH was used as the internal control. The primers applied for cDNA amplification are shown in Table 1.

### 2.6. Quantitation of Cytokines by Cytometric Bead Array

The conditioned media from macrophages and 12Z cells co-culture were collected 24 h after treatment with compounds, centrifuged to remove cellular debris, and applied for subsequent experiments or stored at −80 °C until use. The concentrations of Interleukin-6 (IL-6) and Interleukin 8 (IL-8) in the conditioned media were determined with the Human Inflammatory Cytokine Cytometric Bead Array (CBA) (BD Biosciences, Franklin Lakes, NJ, USA), as previously described [23]. For each kit, a standard calibration curve with maximum and minimum detection limits (1.0–5000 pg/mL) was plotted. Samples were analyzed using a FACSAriaII flow cytometer (BD Biosciences) and FACSDiva v6.1.2 software (BD Biosciences).

### 2.7. Quantitation of Cytokines by ELISA

The monocyte chemoattractant protein-1 (MCP-1) (Biorbyt, Cambridge, UK; sensitivity: 1 pg/mL; specificity: natural and recombinant human CCL2) and tumor necrosis factor-alpha (TNF-α) (R&D Systems, Minneapolis, MN, USA; sensitivity: 0.5–5.5 pg/mL; specificity: natural and recombinant human TNF-α) levels were determined using enzyme-linked immunosorbent assay (ELISA) according to the suppliers’ standard protocols. The prostaglandin E2 (PGE2) concentration was measured using respective ELISA kits (Cayman Chemical, Ann Arbor, MI, USA; sensitivity: 40 pg/mL; specificity: natural PGE2) following the manufacturer’s instructions. The absorbance of reaction mixtures was measured using a Tecan M200 Infnite microplate reader. Results are expressed in pg of cytokine normalized per μg of proteins quantified using Pierce^®^ BCA Protein Assay Kit (Thermo Fisher Scientific, Waltham, MA, USA).

### 2.8. Assessment of Intracellular ROS by Flow Cytometry

Reactive oxygen species (ROS) generation was determined using a CellROX^®^ Deep Red Flow Cytometry Assay Kit (Life Technologies, Carlsbad, CA, USA). In a reduced state, the cell-permeable non-fluorescent CellROX Deep Reagent exhibits a strong fluorogenic signal upon oxidation. The fluorescence intensity of CellROX^®^ Deep Red reflects the ROS levels in live cells. Dead cells were measured using the Sytox^®^ Green Dead Cell Stain (Life Technologies, Carlsbad, CA, USA).

Macrophages were co-cultured with 12Z cells according to standard protocol. After treatment of the co-cultures with resveratrol or its derivatives, macrophages were gently detached from the surface of the flask, rinsed, and centrifuged. Cells were resuspended in RPMI 1640 medium without phenol red, and CellROX^®^ Deep Red reagent was added to the samples at a final concentration of 500 nM and incubated for 60 min at 37 °C in the dark. During the final 15 min of staining, Sytox Green Dead Stain was added in an optimized concentration of 45 nM. Cells were subjected to imaging Amnis™ FlowSight™ flow cytometer (Luminex Corp., Austin, TX, USA) equipped with three lasers for excitation (405, 488, and 642 nm), five fluorescence channels (acquisition by a multi-channel CCD camera), and a side scatter detector. Post-acquisition data analysis was performed using ImageStream Data Exploration and Analysis Software (IDEAS^®^ version 6.2.187, Luminex Corp., Austin, TX, USA). Approximately 0.5 × 10^4^ cells were analyzed in each sample. Single-color compensation controls for CellROX^®^ Deep Reagent and Sytox^®^ Green Dead Stain were also acquired using the integrated software INSPIRE^®^ (version 201.1.0.765; Luminex Corp., Austin, Austin, TX, USA) for data acquisition. The analysis detected oxidatively stressed (live cells), non-stressed cells (live cells), and dead cells representing distinct subpopulations.

### 2.9. Statistical Analysis

The obtained results were expressed as means ± SD from three independent replications. The hypothesis of equality of variances was verified with Levene’s test. Student’s *t*-test was used to compare two groups of data. One-way analysis of variance (ANOVA) followed by Tukey’s post hoc test was applied to determine the differences between the mean values of multiple groups. A statistically significant value was considered at *p* < 0.05; asterisks indicated the level of significance (* *p* < 0.05, ** *p* < 0.01, *** *p* < 0.001). Statistical analysis was performed using STATISTICA version 13.3 software (Statsoft, Inc., Tulsa, OK, USA).

## 3. Results

### 3.1. Differentiation, Polarization of Macrophages, and a Co-Culture of Endometriotic 12Z Cells and M1-Polarized THP-1 Cells

**Figure 3:**
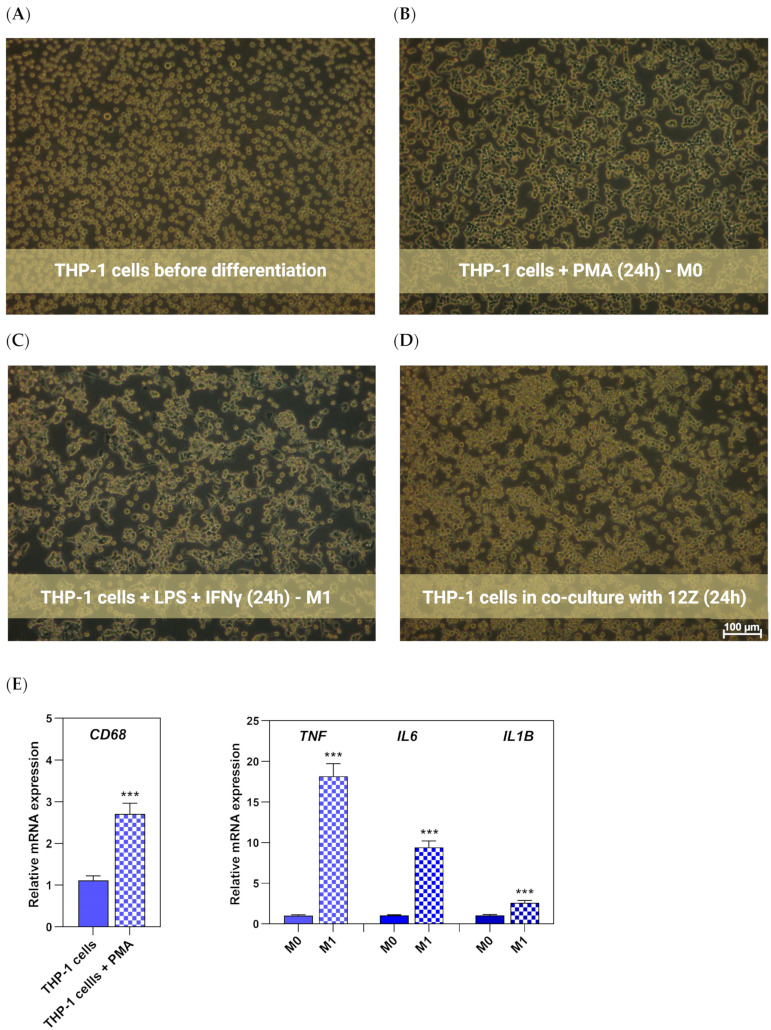
Morphological comparison of THP-1 cells before (**A**) and after PMA induction (**B**) and after M1 polarization (**C**) and co-culture with 12Z (**D**) using a light microscope (magnification × 100). Expression of markers typical for macrophages after differentiation (THP-1 cells + PMA) and macrophages after polarization to M1 phenotype (**E**). Data were normalized against GAPDH and expressed as relative mRNA expression (-fold of control). Statistical analysis assessed differences between exposed cells and negative control with significant differences *** *p* ≤ 0.001.

Human THP-1 monocytes were seeded into the upper compartment of the transwell vessel and subjected to differentiation into macrophages using PMA. As shown in Figure 3A, THP-1 monocytes displayed a suspended culture before induction and were characterized as small, round, and transparent. THP-1 cells exposed to PMA became adherent and larger, a characteristic of M0 cells (Figure 3B). The increase in recognized macrophage marker *CD68* (cluster of differentiation 68), revealed by the qRT-PCR assay, also illustrated the macrophage-like THP-1 (M0 THP-1) phenotype (Figure 3E). Cells acquired a flatter, more spread, and branching morphology M1 phenotype after exposure to LPS and IFN- γ (Figure 3C). As expected, the mRNA expression of *IL1B*, *TNF*, and *IL6* (M1 macrophage markers) was preferentially upregulated (Figure 3E). These results suggest that THP-1 cells were efficiently polarized into M1-like macrophages after PMA and LPS plus IFN-γ induction.

To explore the effects of resveratrol and its analog on the co-culture of polarized THP-1 macrophages and endometriotic 12Z cells, each population was cultured in indirect contact using transwell inserts. Monocytes were seeded onto 0.4 μm pore-size membranes in the inserts, which allowed the exchange of soluble factors and avoided cell transmigration. The monocyte differentiation and then polarization were launched as optimized for monoculture. Polarized THP-1 macrophages were co-cultured with 12Z endometriotic cells in the presence of resveratrol and its analog at different concentrations. At the end of incubation, RNA was extracted from macrophages to measure M1 marker expression, a conditioned medium was collected to quantify the secreted factors, and cells were harvested to measure ROS generation. Macrophages after co-culture with 12Z were presented in Figure 3D.

### 3.2. Effect of Resveratrol and Its Derivatives on 12Z Cell and THP-1-Derived Macrophage Proliferation

**Figure 4:**
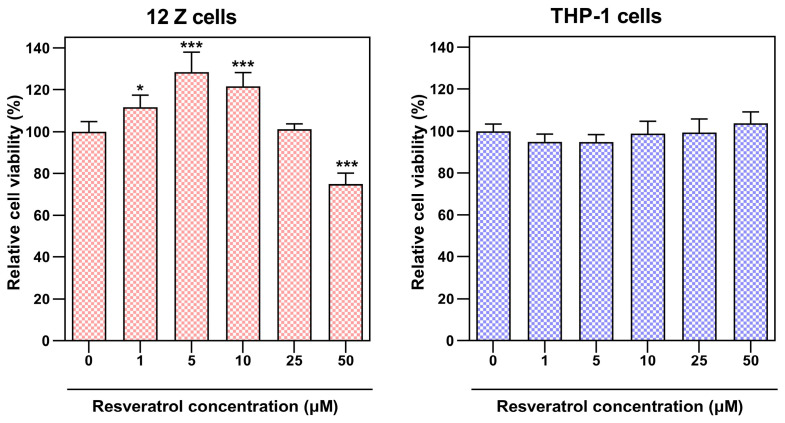
The effect of resveratrol on endometriotic epithelial cell (12Z) and differentiated macrophage (THP-1) proliferation analyzed by MTT reduction assay. The values represent the means (*n* = 6) ± SD. The post hoc Tukey test determined the significance of the effects of resveratrol concentration (* *p* ≤ 0.05; *** *p* ≤ 0.001).

The effect of resveratrol and its natural analogs on the proliferation of endometriotic epithelial 12Z cells and macrophages differentiated from THP-1 monocytes was assessed using the MTT assay. Increasing resveratrol concentration in the endometriotic and macrophage cultures did not cause a suppression of cell proliferation. The compound dosage, responsible for a 20% reduction in viability of 12Z cells, was estimated at 50 µM of compound (Figure 4). The analyzed resveratrol analogs exhibited cytotoxic effects on 12Z cells and THP-1 macrophages comparable to the parent compound. Consequently, we have chosen three dosages: 5, 10, and 25 µM for further studies in the co-culture system.

### 3.3. Effect of Resveratrol and Its Derivatives on the Expression of Genes Related to the Inflammatory and Oxidative Profile of Macrophages Co-Cultured with Endometriotic Cells

**Figure 5:**
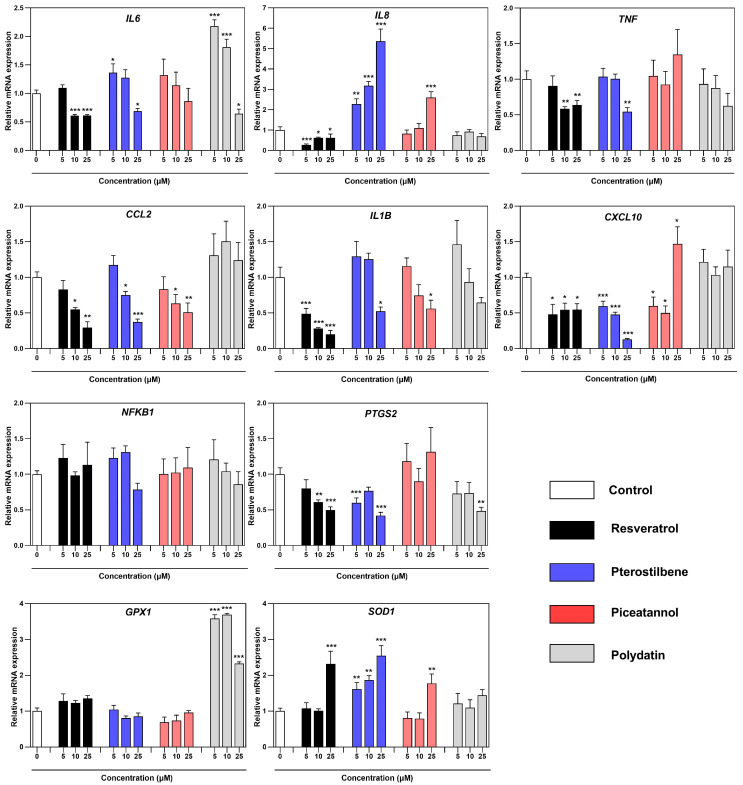
Effect of resveratrol and its analogs on the mRNA expression of *CCL2*, *CXCL10*, *GPX1*, *IL1B*, *IL6*, *IL8*, *NFKB1*, *PTGS2*, *SOD1*, and *TNF* genes in macrophages co-cultured with endometriotic cells, assessed by real-time PCR. Data were normalized against *GAPDH* and expressed as relative mRNA expression (-fold of control). The means ± SD of three independent experiments performed in triplicates are displayed. Statistical analysis assessed differences between the exposed cells and the negative control with significant differences * *p* ≤ 0.05; ** *p* ≤ 0.01; *** *p* ≤ 0.001.

To examine the effect of resveratrol and its derivatives on the macrophages co-cultured with endometriotic cells, qRT-PCR assays were used to analyze the RNA levels of proinflammatory markers *CCL2*, *CXCL10*, *IL1B*, *IL6*, *IL8*, *NFKB1*, and *PTGS2* and endogenous antioxidant enzymes *SOD1* and *GPX1* (Figure 5). Compared to the non-treated, inflamed co-culture model of macrophages and endometriotic cells, in response to resveratrol, we observed the decreased expression of inflammatory markers, such as *IL6*, *IL8*, *IL1B*, *TNF*, *CCL2*, *CXCL10*, and *PTGS2*, mostly at 10 µM and 25 µM concentrations. Especially *CCL2* and *IL1B* showed a significant dose-dependent decrease, reaching 3.5-fold and 5-fold levels at 25 µM concentration, respectively. Pterostilbene and piceatannol revealed a similar capacity to modify the expression of pro-inflammatory markers such as *IL6*, *IL8*, *IL1B*, and *CCL2*. The expression of *CCL2* and *IL1B* also followed a dose-dependent decrease after treatment with pterostilbene and piceatannol. The observed effect of pterostilbene on the down-expression of *CXCL10* was notably pronounced, with the maximum dose reaching 7.7-fold. *IL8* did not follow the pattern of a concentration-dependent decrease of expression after treatment with pterostilbene and piceatannol, whereas only resveratrol induced a significant transcript reduction (up to 3.7-fold). Changes in inflammatory genes related to the treatment under polydatin were not evident, with a reduction only for *IL6* and *PTGS2* at the maximum dose. In the case of the oxidative stress response, the genes encoding *SOD1* showed significant up-regulation at the highest dose of resveratrol, pterostilbene, and piceatannol. The macrophages in the co-culture model treated with the same compounds showed *GPX1* being slightly induced throughout. However, polydatin displayed a high activation pattern and the highest induction (up to 3.7-fold).

### 3.4. Effect of Resveratrol and Its Derivatives on the Cytokine/Chemokine Secretion in an Inflamed Co-Culture Model of Macrophages and Endometriotic Cells

**Figure 6:**
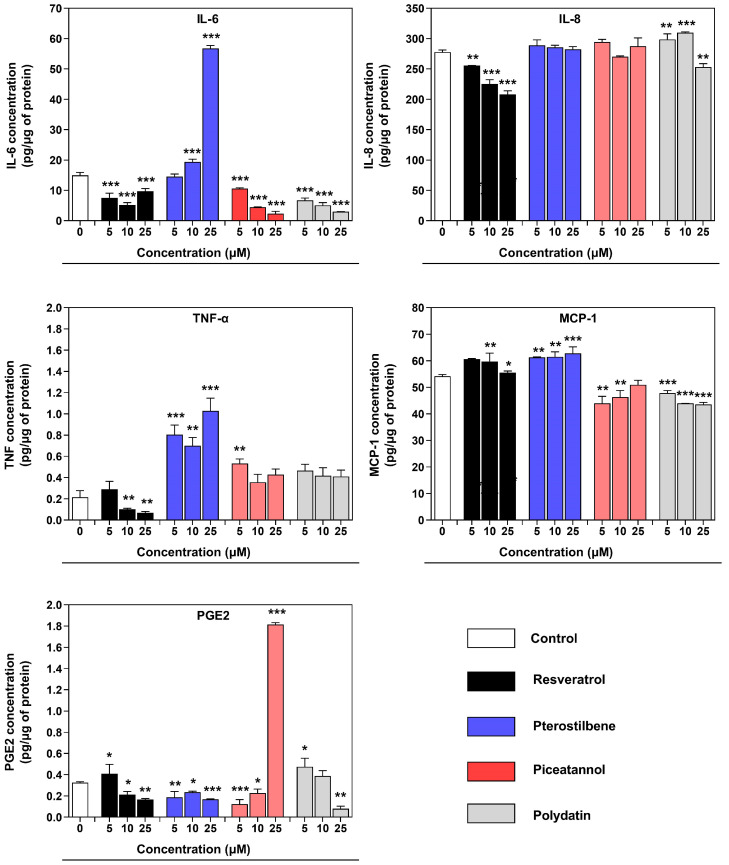
Effect of resveratrol and its analogs on the cytokine/chemokine release in a co-culture model of macrophages and endometriotic cells, assessed by Human Inflammatory Cytokine CBA and ELISA. Data were normalized per μg of proteins in each cell sample. The means ± SD of three independent experiments performed in triplicates are displayed. Statistical analysis assessed differences between the exposed cells and the negative control with significant differences * *p* ≤ 0.05; ** *p* ≤ 0.01; *** *p* ≤ 0.001.

In this experiment, CBA analysis and ELISA assay were used to quantify inflammatory cytokines and chemokines to verify the effect of resveratrol and its analogs on the inflamed co-culture model of macrophages and endometriotic cells. The results for soluble factor quantities calculated per µg of the cell protein are summarized in Figure 6. Compared with the non-treated co-culture model, resveratrol significantly decreased protein levels of IL-6, IL-8, TNF-α, and PGE2 at 10 µM and 25 µM. We detected increased released soluble factors in co-culture supernatants in response to pterostilbene. The increase in IL-6 to 56.8 pg/ug of cell protein was most striking. Only the generation of PGE2 under pterostilbene was significantly reduced at all doses. When piceatannol or polydatin was included in the exposure, the level of IL-6 was markedly decreased. The release of MCP-1 was reduced marginally but significantly in response to piceatannol or polydatin. A noticeable elevated level of PGE2 was detected in the 25 µM piceatannol-exposed co-culture. The exposure to polydatin induced, in turn, a significant reduction in PGE2 quantity in the co-culture supernatant at the highest dose.

### 3.5. Effect of Resveratrol and Its Derivatives on the Intracellular ROS Generation in an Inflamed Co-Culture Model of Macrophages and Endometriotic Cells

**Figure 7:**
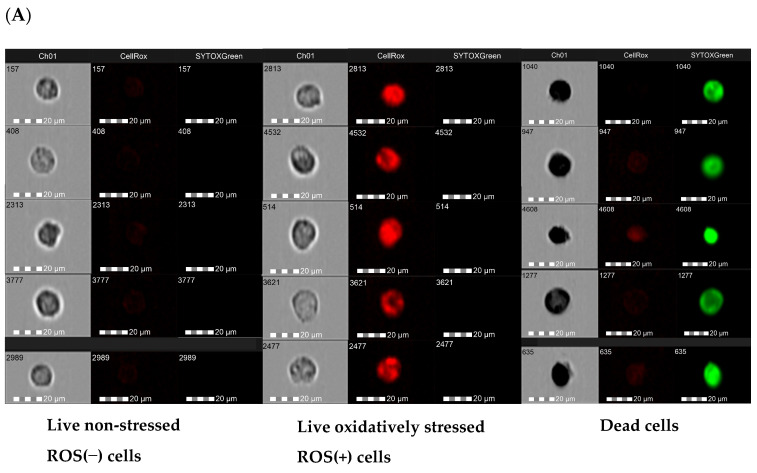

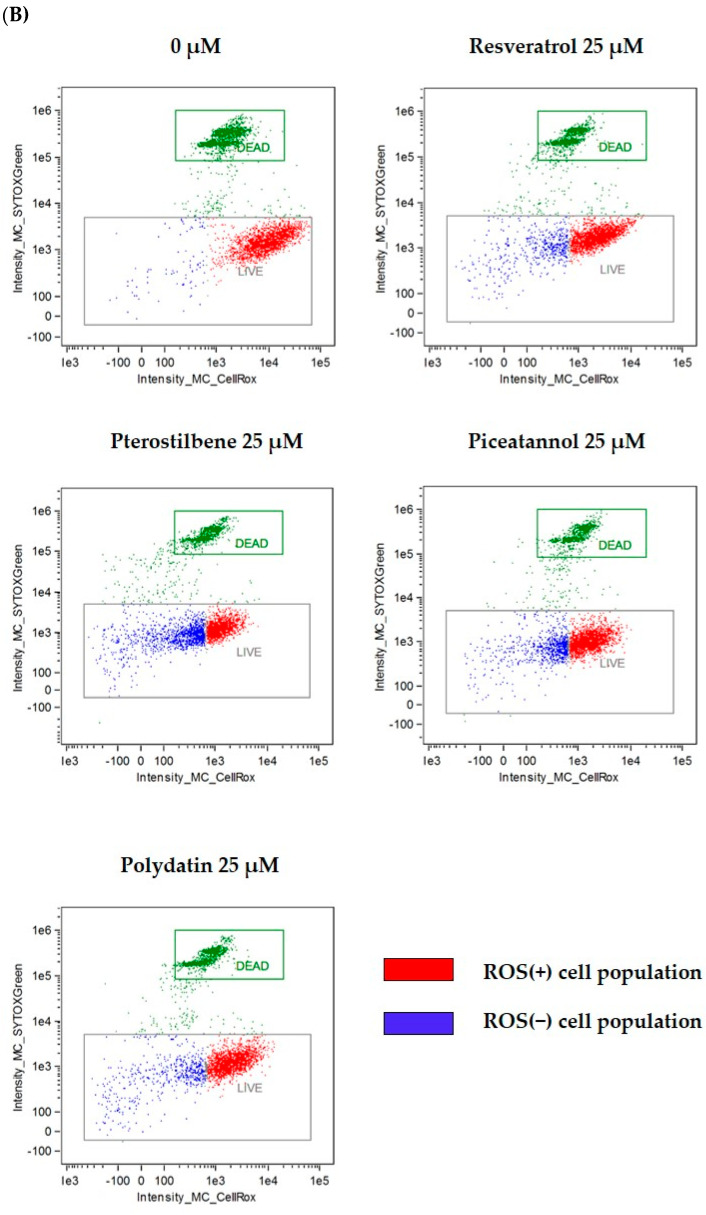

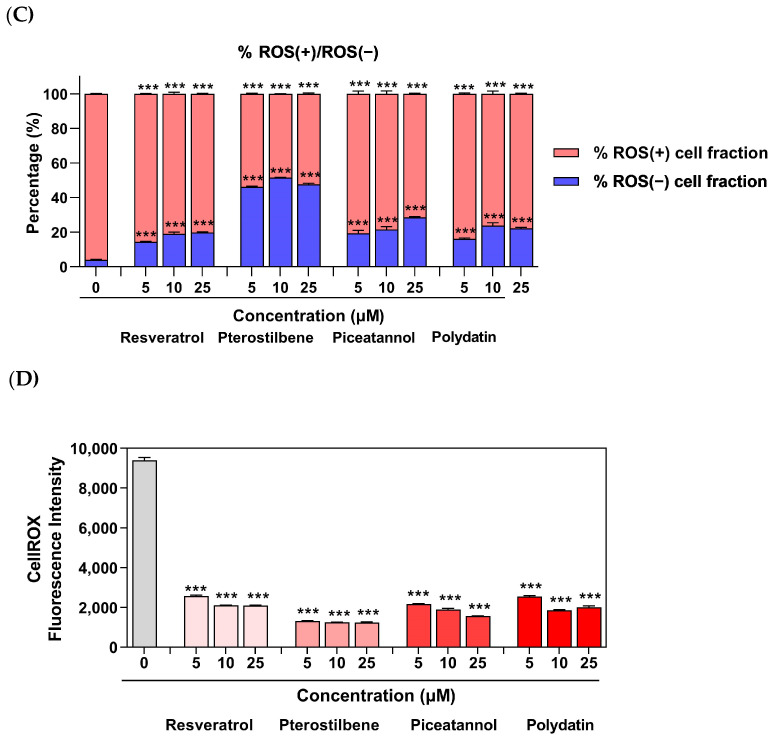
Flow cytometry analysis of macrophages cocultured with 12Z cells stained with CellROX^®^ Deep Red Reagent and Sytox^®^ Green Dead stain. Cells were treated with resveratrol, pterostilbene, piceatannol, polydatin, and their vehicle (0.5% ethanol) for 24 h. (**A**) Representative images of each distinct cellular subset. *n* = 4; scale bars = 20 μm. (**B**) Representative flow cytometry dot plots. (**C**) Summary of CellROX^®^ Deep Red Reagent and Sytox^®^ Green Dead stained populations in macrophages. Data show the percentage distribution of two fractions: live non-stressed cells and live oxidatively stressed cells. (**D**) Quantitative analysis of fluorescence intensity of CellROX^®^ Deep Red. Statistical analysis assessed differences between the exposed cells and the vehicle control with significant differences *** *p* ≤ 0.001.

Intracellular ROS production by macrophages co-cultured with endometriotic cells was examined using CellROX^®^ Deep Red Reagent, a non-fluorescent dye that becomes fluorescent upon oxidation by ROS. Cell viability was monitored using Sytox^®^ Green Dead Stain, which is impermeable to living cells. Double-stained cells were analyzed by flow cytometry to detect two specified parameters: oxidative stress and cell viability. ROS-dependent oxidation and viability of cells were followed in real-time during quantifying by flow cytometry (Figure 7). The CellROX^®^ signal was detected on the internal cell membrane and in the cytosol. The ROS signal was distributed in clusters across different cell parts, eventually merging until the cytosol was fully occupied. Using multiplexed reagents, we differentiated live stressed cells from dead cells; therefore, three distinct sub-populations of cells were specified: live non-stressed cells, live oxidatively stressed cells, and dead cells. Examples of the single-cell images from these sub-populations are presented in Figure 7A. The results in Figure 7B,C show that resveratrol and its analogs dose-dependently increased the population of non-stressed live cells and decreased the sub-population of macrophages producing ROS. Even while exposed to the lowest concentration (5 µM) of all analyzed compounds, a significant rise in the non-stressed population was observed. Pterostilbene was the most effective in reducing the ROS-positive fraction of cells. We assessed ROS levels by determining the fluorescence intensity of CellROX^®^ Deep Red using flow cytometry to investigate the effect of resveratrol and its analogs on ROS generation. Using fluorescence intensity as a read-out of radical production, the highest dose of piceatannol and pterostilbene was found to most effectively prevent ROS production by macrophages by 6- and 7.6-fold, respectively (Figure 7D). Quantification of the fluorescence signal confirmed that all analyzed compounds significantly diminished the accumulation of free radicals.

## 4. Discussion

Endometriosis clinical presentation is often identified with chronic inflammation in the pelvic cavity [24]. Increased monocytes and macrophages in the peritoneal fluid of endometriosis may release a wide range of components, including prostaglandins, cytokines, growth factors, iron, hormones, and enzymes, which are presumed to be the essence of disease initiation and progression [25]. Endometriosis and cancer share a common inflammatory pathogenesis involving TNF-α, IL-1, IL-6, IL-8, and PGE2. These are related to macrophage activation, retrograde menstruation, iron overload, and triggering aberrant inflammatory signaling [26]. In actuality, the dualistic concept of macrophage polarization has been discussed because it may not reflect the dynamic immune environment in vivo [27].

In the peritoneum lesions, there are mixtures of recruited resident and differentiated macrophages; thus, the classification of M1/M2 macrophages may be oversimplified. As it is challenging to replicate the complexity of macrophages using an in vitro cell-based tool, the present study applied proinflammatory phenotypes of monocyte-derived macrophages. To mimic the condition of the inflamed state, a co-culture of endometriotic 12Z cells and macrophages derived from THP-1 was established.

So far, only several in vitro co-culture models have been developed to imitate endometriosis with the immune system represented. To establish immuno-active co-cultures, human endometrial epithelial and stromal cells (HES, HESC), human endometriotic epithelial and stromal cells (12Z, 22B), and THP-1 cells differentiated into macrophages were used. The endometriosis-associated macrophages were macrophages exposed to the conditioned medium obtained from human endometriotic cell cultures [28]. In the studies on the interactions between macrophages and endometriotic epithelial cells, THP-1 cells were differentiated and polarized, and then, 12Z cells were treated with the conditioned media taken from M0, M1, and M2 macrophage cultures [29]. The experimental protocol based on the co-culture of proinflammatory macrophages and endometriotic cells, which was used in this work, allowed for the mutual activation of macrophages and endometriotic cells via soluble factors and enabled the obtaining of more physiologically relevant findings regarding the response of endometriotic microenvironment to anti-inflammatory substances.

The differentiation and polarization protocol for macrophages was optimized in this study, and favorable growth conditions were selected to establish their co-culture with endometriotic cells. The co-culture setup was chosen depending on the doubling rate of the cell line used and the planned length of the experiment, according to the recommendations previously reported [19]. As highlighted, several factors can impact the outcomes of co-cultures with macrophages between research groups, like PMA concentration and differentiation period, LPS and IFN-γ concentration, polarization time, cells’ seeding density, and time of simultaneous co-culture [20,30]. These variabilities might explain different results in the co-culture establishment and systemic response to the treatment.

Our objective in this study was to assess potential differences between resveratrol and its analogs regarding the anti-inflammatory curative potential and resolution of oxidative stress. In studies on tumors, resveratrol has been proven to downregulate the production of pro-inflammatory cytokines, such as TNF-α, IL-6, and IL-1β, and mitigating the inflammatory milieu within the tumor microenvironment hinders the development of angiogenesis, tissue invasion, and metastasis processes [31]. In addition, resveratrol promotes antioxidant defense pathways in several cancer cell lines by eliminating free radicals, enhancing the activities of SOD, CAT, and GPX [17]. As endometriosis behaves malignantly, our study was undertaken to unravel the effect of resveratrol and its analogs in the inflamed model of this disease. The results shown in this article demonstrate that resveratrol and its analogs can suppress the expression of transcript copies of key pro-inflammatory regulators. As already mentioned, not all induced genes followed the same pattern of downregulation depending on the analyzed compound. However, some genes, such as *IL6*, *IL1B*, and *CCL2*, displayed particular responsiveness to resveratrol, pterostilbene, and piceatannol. Resveratrol, pterostilbene, and polydatin, at 25 µM, led to a significant reduction in *IL6* expression. Macrophages in endometriosis produce IL-6, which is implicated in monocyte recruitment and, together with its receptor (IL-6R), regulates endometrial stromal cell growth in vitro [32]. The inhibitory effect on the IL-6 concentration in the co-culture supernatant was observed in our studies after treatment with resveratrol, piceatannol, and polydatin. The outstanding result of the IL-6 concentration after treatment with pterostilbene may be associated with the stimulation effect of endometriotic cells to produce high amounts of IL-6 by 12Z-associated macrophages described in the similar model based on the conditioned media experiments [28]. The immunological role of IL-1β is to stimulate macrophages for the synthesis of IL-6 and activation of COX-2 and enhancement of PGE2 levels, contributing to the synthesis of estradiol by binding of steroidogenic factor 1 (SF-1) to promoters of steroidogenic and aromatase genes [33]. However, the content of this cytokine in the peritoneal fluid of women with endometriosis is still under discussion [32]. The exposure of co-culture to the resveratrol has a prominent effect on reducing *IL1B* expression. The general response of *CCL2* expression remained the same for the resveratrol, pterostilbene, and piceatannol at 10 µM and 25 µM concentrations. Meanwhile, piceatannol and polydatin noticeably reduced their expressions. MCP-1, a well-known chemoattractant cytokine for monocytes/macrophages, is elevated in the peritoneal fluid in women with endometriosis and can act in a paracrine and autocrine on macrophages [32]. The increased level of MCP-1 noted in the co-culture supernatant was probably related to MCP-1 secretion by endometriotic 12Z cells. It was previously reported that MCP-1 secreted from endometriotic epithelial cells recruits macrophages in endometriotic lesions [28]. Another key chemoattractant in endometriosis is IL-8; in our study, *IL8* gene expression and IL-8 protein secretion into co-culture supernatant were reduced only after incubation with resveratrol at all analyzed concentrations.

COX-2, a rate-limiting enzyme in PGE2 production, is overexpressed in endometriotic tissues inducible by inflammation and other pathogenic stimuli. COX-2 is an essential therapeutic target for anti-inflammatory drugs used in treating endometriosis-affected women, which evoke severe side effects such as gastrointestinal, renal, disorders, and cardiovascular toxicity [34]. In light of this, polyphenols may provide alternative nonsteroidal anti-inflammatory drugs because they often are recognized as COX-2 selective [34,35]. Our studies demonstrate that resveratrol, pterostilbene, and polydatin notably reduced COX-2 (*PTGS2*) expression in macrophages co-cultured with endometriotic cells. A qualitatively similar response was observed for the PGE2 concentration in the co-culture supernatant, and the highest concentration of polydatin evoked the most substantial effect.

TNF-α, a product of macrophages, was transcriptionally decreased by the treatment with resveratrol and pterostilbene in our co-culture model. However, the release of this cytokine into the culture medium was blocked only by a maternity compound –resveratrol. Even though TNF-α is a physiological cytokine in the proliferative and secretory-phase endometrium, scientific data indicate a correlation between the concentration of TNF-α in peritoneal fluid and the stage of endometriosis [36].

Resveratrol is a known regulator of inflammatory mediator production through SIRT1 activation. SIRT1, an anti-inflammatory NAD+-dependent deacetylating enzyme, directly deacetylates the RelA/p65 of NF-κB on lysine 310, a crucial site for NF-κB transcriptional activity. Resveratrol potentially enhances the SIRT1 expression and facilitates its attachment to the RelA/p65 substrate. Another important observation revealed that resveratrol blocked NF-κB-regulated cIAP-2 transcription and sensitized cells to TNF-α-induced apoptosis [37]. In several studies, resveratrol treatment blocked nuclear translocation of the p65 subunit of NF-κB in myeloid U-937 cells [38], in TNF-induced prostate cancer PC-3 cells [39], and in human CD34+ cells [40]. Moreover, resveratrol targets include AMP-activated protein kinase (AMPK), whereas AMPK activation increases the NAD+/NADH ratio and triggers its downstream pathways, such as SIRT1 activity [41]. AMPK, a negative regulator of NF-κB, has been reported to be implicated in the inflammatory response in macrophages [42]. SIRT1 plays a direct regulatory role in macrophage functions during inflammation, both in the secretion of cytokines and the expression of cell adhesion molecules as intracellular cell adhesion molecules 1 (ICAM-1) [16]. Several in vitro studies on macrophages have confirmed that resveratrol and its analogs, e.g., piceatannol, upregulate *SIRT1* expression via nuclear translocation of NF-κB [43,44]. In this study, we observed no effect of resveratrol and its derivatives on the expression of the NF-κB transcription factor, a central regulator of the LPS, cytokine, and stress responses in multiple cell types, including macrophages. Studies on the THP-1 cell line have indicated that LPS rapidly induces NF-κB DNA-binding, persists until 12 h of incubation, and is lost entirely after 24 h [45]; therefore, the effect of analyzed compounds in our experiments was not noticeable. Future studies on the NF-κB mechanisms in endometriosis-associated macrophages under treatment with resveratrol and its analogs should clarify the regulation of signaling upstream molecules and their effect on the particular cytokine pathway. Regarding the limitations of in vitro observations, different time checkpoints and cell lines can be included to provide essential insights into the molecular mechanism by which resveratrol suppresses the NF-κB signaling pathway.

Several previous studies have elucidated the impact of resveratrol on the immune response of monocytes and macrophages cultured in vitro. Schwager et al. investigated the resveratrol effect in peripheral blood leukocytes, human umbilical vein endothelial cells, and murine RAW 264.7 and human THP-1 macrophages. Their research presented that resveratrol at a concentration of 25 µM impaired NO production in LPS-activated RAW 264.7 macrophages and exerted significant inhibitory effects on PGE2. Moreover, resveratrol at 25–50 μM markedly reduced the production of IL-6, IL-12(p70), TNF-α, and GM-CSF. At doses as low as 6.25 μM, the effects evoked by resveratrol were more pronounced at the transcription level than protein secretion. The consistent regulation of inflammatory processes was noticed with THP-1 cells, which indicated no species-specific relationships. At 25 μM, resveratrol almost entirely dampened the PGE2 production in LPS-activated THP-1 cells. Furthermore, IL-1β, IL-6, and *CXCL10*/IP-10 were significantly reduced. Regarding gene expression regulation, *IL1B*, *IL6*, *TNF*, and chemokines, including *CCL2* and *CXCL10*, were clearly down-regulated. Interestingly, similarly to our studies, the expression level of *CXCL8* was not decreased by resveratrol treatment. It should be noted that, in the research reported, RNA was extracted from THP-1 cells after 4 h of activation, and cell culture supernatants were recovered after 24 h [46]. Likewise, in another study, resveratrol (5–25 μM) pretreatments markedly reduced *IL6* and *PTGS2* mRNA levels in LPS-stimulated cells but did not influence *IL1B* and *CXCL8* transcripts. The increased IL-6 protein secretion by LPS-treated differentiated THP-1 cells was also lowered by treatment with resveratrol at concentrations ranging from 10 to 25 μM. Moreover, the inhibitory effect of pterostilbene was documented in the differentiated LPS-induced THP-1 macrophages, in which IL-6 was down-regulated at the mRNA and protein levels [47].

Several promising and consistent in vitro studies have shown that resveratrol has beneficial effects on the mono-culture of endometriotic cells regarding its anti-inflammatory properties. Kolahdouz-Mohammadi et al. were the first to describe the inhibitory effect of resveratrol therapy on MCP-1, IL-6, IL-8, and RANTES expression in ectopic endometrial stromal cells of endometriotic women [48]. Similar studies with endometriotic stromal cells demonstrated that resveratrol treatment significantly suppressed TNF-α-induced IL-8 release through endogenous SIRT1 activation [49]. However, the significant effects of resveratrol and its analogs on the inflammatory pathology in endometriosis have been described for the first time in the present work. The use of endometriotic cells together with macrophages underlined the importance of establishing a complex in vitro model, which addresses interactions between cells in the endometriotic microenvironment. The obtained results expand current knowledge about the anti-inflammatory properties of the stilbenes, examined on the mono-culture of endometriotic cells or immune cells.

Macrophages, erythrocytes, and apoptotic endometriotic cells are found to induce oxidative stress in endometriosis. ROS and pro-inflammatory factors are connected, contributing to pain and the failure to detoxify lipid peroxidase products under oxidative stress. Pro-inflammatory and chemotactic cytokines play a significant role in the recruitment and activation of phagocytic cells, which are the leading producers of ROS [50]. With the deepening of oxidative stress in the pelvic cavity, harmful damage, like tissue injury and chronic inflammation, is beginning to appear, resulting in the proliferation and fibrosis of ectopic endometrial lesions [33]. Our findings indicate that all analyzed stilbenes significantly reduced ROS accumulation in macrophages co-cultured with 12Z cells. A reduced fraction of ROS-positive cells was detected even with the treatment of the lowest concentration of the stilbenes (5 µM). It is worth emphasizing that the reduction in cells excessively producing ROS was observed under pterostilbene treatment by about 50%. These findings are correlated with the increasing expression of the antioxidant enzyme SOD1 in macrophages. These results are consistent with evidence suggesting that aberrant changes in endogenous antioxidant enzymes SOD as the first line of defense against oxygen free radicals and GPX—a major peroxide-scavenging enzyme—can contribute to the oxidative damage in endometriosis and be proposed as a biomarker for endometriosis [51,52].

Like other model in vitro experiments, our studies have some limitations that should be pointed out when discussing the findings. First, in the experimental system, we included two types of cell lines. However, the endometriosis niche is known to exist in a complex environment with a dynamic population of epithelial, stromal, immune, endothelial, and glandular cells [53]. On the other hand, validating the specific pathology, like altered inflammatory processes in the disease, mostly requires using cells, which are the most committed. Secondly, only one phenotype of macrophages was used to form co-culture with endometriotic cells. To validate the present results more significantly, further studies involving naive (M0) and alternatively activated (M2) macrophage populations are needed to represent a more comprehensive immunodeficiency manifestation of the disease. Additionally, which phenotype of macrophages is responsible for disease progression is still debated. Some analyses highlight that the pro-inflammatory macrophage population mainly occurs in the early stages of the disease, whereas the switch to M2-polarization appears in advanced stages together with pro-fibrotic activity [54,55]. The point that is often emphasized is that many physiological or pathological macrophages do not show a clear M1 or M2 phenotype, and using the terms M1 and M2 with a specific phenotypic scoring criterion is confusing. Moreover, in that type of study, the verification of properties of natural compounds should be addressed to improve the bioaccessibility of the drug by considering their gastrointestinal digestion and metabolism by gut microorganisms [6]. Therefore, in our experiments, we investigated the immunomodulatory effects of resveratrol and its derivatives at low physiologically attainable concentrations, based on the last work of Feng et al., who observed in vitro and in vivo physiological (5 µM) and pharmacological (>10 µM) effects of resveratrol on THP-1 cells and rodent model [47,56].

## 5. Conclusions

Summarizing our findings and considering the conclusions from the reported studies on inflamed co-culture of pro-inflammatory macrophages and endometriotic cells, we can state that resveratrol and its analogs can target immune dysregulation and oxidative imbalance in endometriosis. Most of the observed changes in gene expression, cytokine/chemokine concertation, and real-time production of ROS support this hypothesis, with some notable exceptions. Differences in the anti-inflammatory and antioxidant efficiency of the compounds confirmed that each stilbene can act preferentially on specific pathways in the cell. Our findings may open the way to evaluating alternative therapy and dietary support in endometriosis treatment and alleviating endometriosis-related disorders. Optimally designed functional food products enriched with stilbenes could be a valuable source of these compounds in the daily diet of women with endometriosis. However, further extended pre-clinical studies are necessary to provide strong evidence of resveratrol and its analogs’ therapeutic potential. The research should be programmed to develop optimal stilbene delivery methods and improve their bioavailability, increasing effectivity and decreasing efficient administration doses [11,14]. In vitro studies on human cells provide data on the potential activity and predicted effectivity of natural compounds that may enhance their relevance and applicability in further extensive research. Despite promising results indicating that resveratrol and its analogs attenuate immunopathology and oxidative stress in endometriosis [57], these data cannot be directly translated into clinical effects. In addition, several recent clinical trials revealed the positive use of resveratrol as an adjuvant treatment against, e.g., endometriotic chronic pelvic pain [58,59]. Therefore, well-controlled further clinical trials are absolutely required to evaluate the therapeutic potential of stilbenes administered as functional food products, dietary supplements, or drug formulations.

## Figures and Tables

**Table 1:** Primer sequences used in real-time PCR.

Gene	Accession	No. Sequence (5′-3′)	Amplicon (bp)
*CCL2*	NM_002982.4	F: AGAATCACCAGCAGCAAGTGTCC R: TCCTGAACCCACTTCTGCTTGG	98
*CXCL10*	NM_001565.4	F: GAACTGTACGCTGTACCTGCA R: TTGATGGCCTTCGATTCTGGA	172
*GAPDH*	NM_002046.7	F: GTCTCCTCTGACTTCAACAGCG R:ACCACCCTGTTGCTGTAGCCAA	131
*GPX1*	NM_000581.4	F: GTGCTCGGCTTCCCGTGCAAC R: CTCGAAGAGCATGAAGTTGGGC	123
*IL1B*	NM_000576.3	F: CCACAGACCTTCCAGGAGAATG R: GTGCAGTTCAGTGATCGTACAGG	131
*IL6*	NM_000600.5	F: AGACAGCCACTCACCTCTTCAG R: TTCTGCCAGTGCCTCTTTGCTG	132
*IL8*	NM_001354840.3	F: GAGAGTGATTGAGAGTGGACCAC R: CACAACCCTCTGCACCCAGTTT	112
*NFKB1*	NM_003998.4	F: GCAGCACTACTTCTTGACCACC R: TCTGCTCCTGAGCATTGACGTC	130
*PTGS2*	NM_000963.4	F: CGGTGAAACTCTGGCTAGACAG R: GCAAACCGTAGATGCTCAGGGA	156
*SOD1*	NM_000454.5	F: CTCACTCTCAGGAGACCATTGC R: CCACAAGCCAAACGACTTCCAG	129
*TNF*	NM_000594.4	F: CTCTTCTGCCTGCTGCACTTTG R: ATGGGCTACAGGCTTGTCACTC	135

## Data Availability

The raw data supporting the conclusions of this article will be made available by the authors upon request.
